# Surgery for bacterial endocarditis associated with bacteria of oral/dental origin – characteristics of the “typical host” and implications for prevention

**DOI:** 10.1186/1749-8090-8-S2-P1

**Published:** 2013-10-23

**Authors:** A Borowski, J Eckert, H Dalyanoglu, E Godehardt

**Affiliations:** 1 Cardiovascular Surgery, University of Duesseldorf, Germany

## Background

To characterize the “typical host” for bacterial endocarditis (BE) of oral/dental origin and to address possible implications for prevention.

## Methods

104 patients who underwent surgery for BE were divided into two groups according to the presumable port of entry, Group A (n=14) with bacteria of oral flora (“oral entry port”), and for comparison, Group B (n=90) with bacteria of non-oral origin (“non-oral entry port”). Risk profile, clinical course and outcome were assessed.

## Results

Significant differences between the groups were found regarding poor oral health, metabolic syndrome and dental treatment with a higher incidence in Group A. In Group A, majority of cases had left-sided endocarditis (79%); in Group B, 63% of patients were diagnosed with left-sided-, 30% with left-and-right-sided-, and 7% with right-sided-endocarditis. The in-hospital mortality was 0% vs. 26% in Group A and Group B, respectively.

## Conclusions

The findings of our study suggest that BE associated with pathogens of oral flora mainly affects left-sided native heart valves, and the typical host is a patient with metabolic syndrome and poor dental status. Dentists should be alert in dealing with these patients in terms of a continuous preventive and therapeutic measure to maintain their optimal oral health.

